# Inflammation-based scores as predictors of treatment response in advanced adrenocortical carcinoma

**DOI:** 10.1530/ERC-22-0372

**Published:** 2023-03-15

**Authors:** Alessandra Mangone, Barbara Altieri, Mario Detomas, Alessandro Prete, Haider Abbas, Miriam Asia, Yasir S Elhassan, Giovanna Mantovani, Cristina L Ronchi

**Affiliations:** 1 Department of Clinical Sciences and Community Health, University of Milan, Milan, Italy; 2 Institute of Metabolism and Systems Research, University of Birmingham, Birmingham, UK; 3 Division of Endocrinology and Diabetes, Department of Internal Medicine I, University Hospital, University of Würzburg, Würzburg, Germany; 4 Centre for Endocrinology, Diabetes and Metabolism, Birmingham Health Partners, Birmingham, UK; 5 Department of Endocrinology, Queen Elizabeth Hospital Birmingham, University Hospitals Birmingham NHS Foundation Trust, Birmingham, UK; 6 Oncology Department, Queen Elizabeth Hospital Birmingham, University Hospitals Birmingham NHS Foundation Trust, Birmingham, UK; 7 Endocrinology Unit, Fondazione IRCCS Ca' Granda Ospedale Maggiore Policlinico, Milan, Italy

**Keywords:** adrenal cancer, prognosis, chemotherapy, mitotane, neutrophil–lymphocyte ratio, platinum, EDP

## Abstract

Treatment for advanced adrenocortical carcinoma (ACC) consists of mitotane alone or combined with etoposide, doxorubicin, and cisplatin (EDP). Although both therapies are widely used, markers of response are still lacking. Since inflammation-based scores have been proposed as prognostic factors in ACC, we aimed to investigate their role in predicting the response to first-line chemotherapy.

We performed a retrospective analysis of patients with advanced ACC treated with mitotane monotherapy or EDP ± mitotane. Clinical parameters (tumour stage at diagnosis, resection status, Ki67, time from diagnosis to treatment start, performance status, plasma mitotane levels, time in mitotane target ≥ 80%, clinically overt cortisol hypersecretion), and pretreatment inflammation-based scores (neutrophil-to-lymphocyte ratio (NLR), platelet-to-lymphocyte ratio (PLR), monocyte-to-lymphocyte ratio, derived neutrophil-to-lymphocyte ratio) were investigated. The primary endpoints were overall survival (OS) and time-to-progression (TTP) from treatment initiation, the secondary endpoint was the best objective response to treatment.

We included 90 patients (59% = women, median age = 51 years) treated with mitotane monotherapy (*n* = 40) or EDP ± mitotane (*n* = 50). In the mitotane monotherapy cohort, NLR ≥ 5 and PLR ≥ 190 predicted shorter OS (hazard ratio (HR): 145.83, 95% CI: 1.87–11,323.83; HR: 165.50, 95% CI: 1.76–15,538.04, respectively), remaining significant at multivariable analysis including clinical variables. NLR was also associated with shorter TTP (HR: 2.58, 95% CI: 1.28–5.20), but only at univariable analysis. Patients with NLR ≥ 5 showed a worse treatment response than those with NLR < 5 (*P* = 0.040). In the EDP ± mitotane cohort, NLR ≥ 5 predicted shorter OS (HR: 2.52, 95% CI: 1.30–4.88) and TTP (HR: 1.95, 95% CI: 1.04–3.66) at univariable analysis.

In conclusion, inflammation-based scores, calculated from routinely measured parameters, may help predict response to chemotherapy in advanced ACC.

## Introduction

Adrenocortical carcinoma (ACC) is a rare malignancy with an incidence of 0.5–2/million persons/year (Kerkhofs *et al.* 2013, Else *et al.* 2014, Fassnacht *et al.* 2018). Although clinical management of ACC has improved over the years, its prognosis remains largely unfavourable. Radical surgical resection is the most effective strategy for localized tumours, but recurrence is common even after a margin-negative resection (Elhassan *et al.* 2021, Shariq & McKenzie 2021). Moreover, approximately half of the patients have metastatic or inoperable disease at diagnosis (Shariq & McKenzie 2021). For patients with advanced ACC, mitotane represents the current standard of care, used as monotherapy for selected patients with low tumour burden and/or slower growth rate, or in combination with etoposide, doxorubicin, and cisplatin (EDP) chemotherapy (Fassnacht *et al.* 2018, Fassnacht *et al.* 2020). Nevertheless, mitotane with or without EDP is associated with a relatively limited response rate and is burdened with important toxicity (Fassnacht *et al.* 2012, Megerle *et al.* 2018, Fassnacht *et al.* 2020). Although multiple parameters have been shown to be relevant for the prognostic classification of ACC (Elhassan *et al.* 2021), reliable predictors of treatment outcomes in advanced disease are still lacking (Roca *et al.* 2017, Laufs *et al.* 2018, Altieri *et al.* 2020, Altieri *et al.* 2022). Such predictors would allow the delivery of a more personalized approach, aiming to prioritize the quality of life of affected individuals (Steenaard *et al.* 2019).

The role of chronic inflammation in the development and progression of multiple cancers is a topic of increasing interest (Coussens & Werb 2002, Mantovani *et al.* 2008), and an independent prognostic role of peripheral blood-derived inflammation-based scores has been demonstrated in different cancer types. Among the most evaluated, neutrophil-to-lymphocyte ratio (NLR), derived neutrophil-to-lymphocyte ratio (dNLR), platelet-to-lymphocyte ratio (PLR), monocyte-to-lymphocyte ratio (MLR), serum albumin, and Glasgow prognostic score have shown an association with survival rates in a variety of cancers (Forrest *et al.* 2005, Templeton *et al.* 2014, Yodying *et al.* 2016, Capone *et al.* 2018, Zhang *et al.* 2021). The best score and optimal cut-off value vary depending on the cancer type and patients’ characteristics (e.g. ethnical differences) (Azab *et al.* 2014, Dolan *et al.* 2017), and there is high heterogeneity in the thresholds used (Dupré & Malik 2018). Since most ACCs are associated with autonomous cortisol secretion and immune cell count correlates with the degree of hypercortisolism (Detomas *et al.* 2022), an inflammation-based score may play an even more relevant role in the prognostic stratification of this aggressive cancer. Previous studies have evaluated the prognostic significance of pre-operative inflammation-based scores in patients with ACC undergoing surgery, showing promising results in predicting the diagnosis of primary adrenal malignancies and clinical outcomes, although in small cohorts (Bagante *et al.* 2015, Mochizuki *et al.* 2017, Gaitanidis *et al.* 2019, Sisman *et al.* 2020, de Jong *et al.* 2021, Solak *et al.* 2021, Detomas *et al.* 2022). Recently, Grisanti *et al.* also showed the role of these biomarkers as predictive factors of response to gemcitabine plus capecitabine chemotherapy, currently regarded as a second-line scheme in progressive ACC (Grisanti *et al.* 2021).

We, therefore, aimed to investigate the role of inflammation-based scores in predicting response to first-line chemotherapy, including mitotane as monotherapy or combined with EDP, in patients with advanced ACC.

## Subjects and methods

### Patients’ selection

Retrospective analysis of available clinical data of adults (≥ 18 years) with a documented diagnosis of ACC followed up at Queen Elizabeth Hospital Birmingham (UK) or University Hospital of Würzburg (Germany) between 2005 and July 2022. We then selected patients with advanced or progressive disease (PD) treated with mitotane monotherapy or EDP ± mitotane for whom white blood cell differential (WBCD) count was available at the time of initiation of the treatment of interest. Advanced ACC was defined as non-resectable or metastatic disease based on radiological findings. Other inclusion criteria were as follows: available radiological data during follow-up; Eastern Cooperative Oncology Group (ECOG) performance status 0–2; adequate liver and kidney function; at least 2 months of continuous mitotane treatment or one cycle of EDP. There were no differences in the EDP schemes between the two centres (i.e. initial doses, interval between cycles and dose titration according to the FIRM-ACT study (Fassnacht *et al.* 2012). We excluded patients with sepsis and other known infections at the time of blood testing, severe haematological diseases, active malignancies other than ACC, severe cardiopathy, active autoimmune disease, or treatment with glucocorticoids or other immunomodulatory drugs. The study was approved by the hospitals’ local ethics committees (Birmingham HBRC 11/606 and Prime-Act, Wuerzburg 88/11), and all patients provided written informed consent.

### Treatment details

The treatment approach to all cases was discussed in specialist multidisciplinary meetings. In accordance with the current European guidelines (Fassnacht *et al.* 2018, Fassnacht *et al.* 2020), patients with advanced ACC at the time of diagnosis not qualifying for local treatment or with recurrence occurring less than 6 months after surgery were treated with mitotane monotherapy or EDP+mitotane. Mitotane monotherapy was generally used in patients with lower tumour burden or more indolent disease, whilst in the case of disseminated disease EDP was chosen. Patients were treated until significant disease progression, intolerable toxicity, and/or further multidisciplinary team treatment decision. Tumour assessment was performed at baseline, at week 12, and every 8–12 weeks thereafter, and clinical responses were classified according to response evaluation criteria in solid tumours – RECIST 1.1 (Eisenhauer *et al.* 2009). Periodical imaging included thorax–abdomen–pelvis computed tomography (CT) with contrast, nuclear magnetic resonance, or fluorodeoxyglucose (FDG)-positron emission tomography/CT scan.

### Data collection

Clinical parameters were collected at the time of diagnosis, before the initiation of systemic therapy, and during follow-up. Specifically, we considered the tumour stage at diagnosis, in accordance with the European Network for Study of Adrenal Tumours (ENSAT) (Fassnacht *et al.* 2009), resection (R) status of the primary tumour, Ki67 index, time from diagnosis to treatment initiation, ECOG performance status, plasma mitotane levels, time in plasma mitotane target ≥ 80%, and evidence of clinically overt cortisol hypersecretion. Inflammation-based scores were collected before the initiation of the treatment of interest, although some patients were already receiving mitotane (i.e. as adjuvant therapy when disease recurrence was recorded, or underwent treatment escalation from mitotane alone to EDP+mitotane due to disease progression, Table 1). Pre-treatment inflammation-based scores were calculated starting from serum albumin and WBCD count, as follows: NLR, dNLR, PLR, and MLR were calculated as neutrophil count divided by lymphocyte count, neutrophil count divided by (white blood cell count − neutrophil count), platelet count divided by lymphocyte count, and monocyte count divided by lymphocyte count, respectively.

**Table 1:** Demographic and clinical data of 90 patients with advanced adrenocortical carcinoma (ACC) at start of palliative therapy.

At start of palliative therapy	Mitotane cohort	EDP cohort	*P* value
Cases, *n*	40	50^a^	
Sex, F/M (% F)	24/16 (60)	29/21 (58)	1.000
Median age (range), years	54 (23–84)	51 (20–77)	0.092
Symptoms related to ACC (%)	14/35 (40)	27/49 (55)	0.191
Unknown	5/40	1/50	
Cortisol hypersecretion	11/34 (32)	25/49 (51)	0.1167
Unknown	6/40	1/50	
Time from diagnosis to therapy			
Median time (range), months	14 (0.5–150)	4 (0.5–61)	**0.032**
≤ 6 months (%)	17/40 (42)	31/50 (62)	0.089
> 6 months (%)	23/40 (58)	19/50 (38)	
Mitotane therapy already ongoing^b^ (%)	12/40 (30)	24/50 (48)	0.129
Mitotane ≥ 80% of time at range (%)	6/12 (50)	11/24 (46)	1.000
Mitotane concentration mean ± s.d., mg/L	15.6 ± 6.3	13.7 ± 9.2	0.556
ECOG performance status			
0 (%)	23/38 (60)	19/42 (45)	**0.020**
≥ 1 (%)	15/38 (40)	23/42 (55)	
Unknown	2/40	8/50	

^a^Forty-three patients received EDP + mitotane, and seven patients received EDP without mitotane; ^b^Mitotane therapy already ongoing as adjuvant therapy in the mitotane cohort and as adjuvant/palliative therapy in the EDP cohort when the first evidence of disease recurrence or progression was noted. Significant *P* values are bold. ACC, adrenocortical carcinoma; ECOG, Eastern Cooperative Oncology Group; EDP, etoposide, doxorubicin, and cisplatin; F, female; M, male; N, number.

### Study endpoints

Primary endpoints were overall survival (OS), defined as the interval from the initiation of treatment to death or last follow-up visit, and time-to-progression (TTP), defined as the time elapsed from treatment initiation to the first radiological evidence of disease progression or death. Secondary endpoint was the response to therapy, defined as the best response during treatment administration based on RECIST criteria (Eisenhauer *et al.* 2009): PD, stable disease (SD), partial response (PR), mixed response (MR), and complete response (CR). Clinical benefit was defined as SD + PR + CR + MR, as previously described (Grisanti *et al.* 2021).

### Statistical analysis

Descriptive statistics were used to report clinical indicators. For quantitative variables, comparisons were carried out using non-parametric Wilcoxon test. Associations between categorical variables were assessed by Chi-square or Fisher exact tests as appropriate.

For survival analysis, the cut-offs used for some WBDC-derived parameters were obtained from current literature on ACC: NLR = 5, PLR = 190, albumin = 39 (Bagante *et al.* 2015, Mochizuki *et al.* 2017, Grisanti *et al.* 2021, Zhang *et al.* 2021). With respect to MLR and dNLR, which have not yet been analyzed in this type of cancer, we used the median value observed in our cohort as the cut-off (0.4 and 2.5, respectively). Established prognostic variables including ENSAT tumour stage (Fassnacht *et al.* 2009), R status (Bilimoria *et al.* 2008), Ki67 index (Beuschlein *et al.* 2015), ECOG performance status (Grisanti *et al.* 2021), clinically overt cortisol hypersecretion (Vanbrabant *et al.* 2018), and time in mitotane target (Puglisi *et al.* 2020) were also tested in survival analysis. Survival curves were generated with the Kaplan–Meier method, and outcomes were compared with Cox regression analysis. Clinical variables with a potential prognostic value at univariable Cox regression (enter level *P* ≤ 0.1) were included in a multivariable Cox model. Results are given as hazard ratio (HR) with 95% confidence intervals (95% CIs). The statistical significance was conventionally set at *P* < 0.05. All analyses were performed with SPSS version 28 (IBM) and GraphPad Prism version 9 (GraphPad Software).

## Results

### Patients’ characteristics

Ninety patients with advanced ACC were included in the study, subdivided into two cohorts depending on the treatment received: mitotane monotherapy (mitotane cohort, *n* = 40) and EDP ± mitotane (EDP cohort, *n* = 50). Treatment escalation from mitotane alone to EDP+M due to disease progression was undertaken in 15 patients who were therefore included in both groups. In the EDP cohort, 43 patients were treated with EDP+mitotane, while 7 patients received EDP without mitotane for previous toxicity.

The demographics and clinical data of the patients included in the study are detailed in Table 1, reporting data at treatment start, and in Supplementary Table 1 (see section on supplementary materials given at the end of this article), reporting data of patients at initial diagnosis. The median age at treatment initiation was 54 years in the mitotane cohort (range 23–84) and 51 years in the EDP cohort (range 20–77), with 60 and 58% women, respectively. Overall, 36 (40%) patients had metastatic disease at the time of diagnosis (ENSAT stage IV: 30% of patients in the mitotane cohort and 48% of those in the EDP cohort). Surgery was not performed in 20% of patients because of the metastatic disease, whilst the rest underwent resection of the primary tumour with curative or palliative intent, which was radical (R0) in 40% of cases. The median Ki67 proliferation index was 20 and 27% in the mitotane and EDP cohort, respectively. The median time from diagnosis to initiation of therapy was 14 months in the mitotane and 4 months in the EDP cohort. Overall, 36/90 patients were already receiving mitotane therapy when the first evidence of disease recurrence or progression was noted. Before starting therapy, approximately half of the patients (40 and 55% in the mitotane and EDP group, respectively) had an ECOG performance score ≥ 1; clinically overt cortisol hypersecretion was present in 32% and 51% of patients in the mitotane and EDP cohort, respectively. The median duration of palliative mitotane treatment was 6 months (range 2–132), and a median number of 3.5 cycles (range 1–8) of EDP was administered.

### Response to systemic treatment

The survival analyses and treatment efficacy in the two cohorts are reported in Table 2. Only 18 (20%) patients were still alive at the end of the follow-up period. The median OS from the start of considered treatment was 16 (range 3–133) and 10 months (range 1–82), while TTP was 4 (range 1–96) and 3 months (range 1–8) in the mitotane and EDP cohort, respectively. Median OS and median TTP from therapy initiation were significantly shorter (*P* = 0.008 and 0.012, respectively) in the EDP vs mitotane cohort (as expected considering that mitotane monotherapy is usually given to selected patients with low volume or indolent disease). As shown in Table 2, in the mitotane cohort, 25 (62.5%) patients had PD, while 15 (37.5%) patients experienced a clinical benefit. In the EDP cohort, 20 (40%) patients showed progression and 30 (60%) clinical benefit (*P* = 0.041).

**Table 2:** Follow-up data and response to therapy in the two cohorts of patients with advanced adrenocortical carcinoma (*n* = 90).

Endpoints of survival and response to therapy	Mitotane cohort	EDP cohort	*P* value
Median overall survival from diagnosis (range), months	37 (4–156)	20 (2–98)	**0.006**
Dead at last follow-up (%)	29/40 (73)	43/50 (86)	0.122
Median overall survival from start of therapy (range), months	16 (3–133)	10 (1–82)	**0.008**
Median time to progression from start of therapy (range), months	4 (1–96)	3 (1–8)	**0.012**
Best objective response to therapy			**0.041**
Progressive disease (%)	25/40 (62.5)	20/50 (40)	
Stable disease (SD) (%)	8/40 (20)	16/50 (32)	
Partial response (PR) (%)	5/40 (12.5)	12/50 (24)	
Complete response (CR) (%)	2/40 (5)	0/50 (0)	
Mixed response (MR) (%)	0/40 (0)	2/50 (4)	
SD + PR + CR + MR	15/40 (37.5)	30/50 (60)	**0.056**

Significant *P* values are bold. EDP, etoposide, doxorubicin, and cisplatin.

In the EDP cohort, 13 (26%) patients required a reduction of the dosage of etoposide and/or doxorubicin or discontinuation of treatment because of chemotherapy-related adverse events, mainly neutropoenia.

### Predictive role of inflammation-based scores

**Figure 1:**
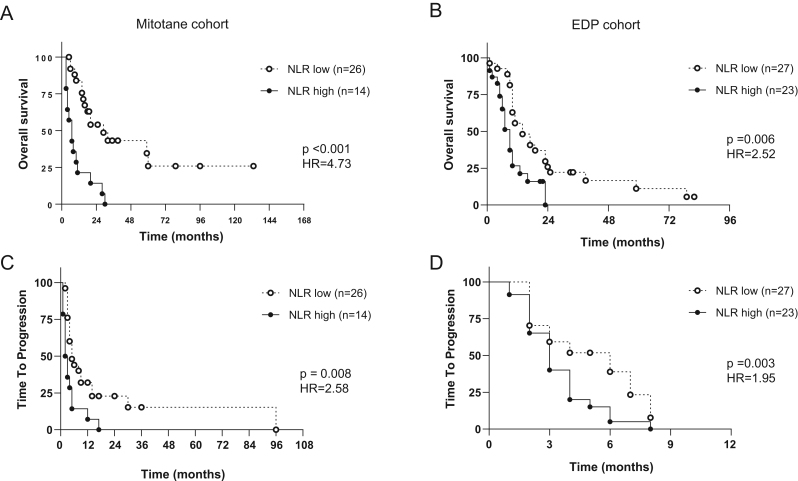
Kaplan–Maier curves for overall survival (OS) and time-to-progression (TTP) from the start of treatment for patients treated with mitotane monotherapy (A and C, *n* = 40) or EDP ± mitotane (B and D, *n* = 50). EDP, etoposide, doxorubicin and cisplatin; HR, hazard ratio; NLR, neutrophil-to-lymphocyte ratio. Patients were stratified according to pre-treatment NLR in NLR low, dashed line = NLR < 5; NLR high, black line = NLR ≥ 5. Comparisons between survival curves were performed with Cox regression.

Survival analyses were performed separately in the two cohorts, evaluating both traditional prognostic clinical and histopathological variables as well as inflammation-based scores. NLR was the strongest predictor of the primary and secondary endpoints in both cohorts (see Fig. 1 for Kaplan–Maier curves for OS and TTP).

In the mitotane cohort, NLR, dNLR, PLR, and MLR together with R-status, time from diagnosis to initiation of treatment, ECOG performance status, cortisol hypersecretion, and time in mitotane target were strongly associated with OS at univariable analysis, while there was no significant association with albumin and initial ENSAT stage or Ki67 (Table 3). At multivariable analysis, only NLR (HR: 145.83, 95% CI: 1.87–11,323.83, *P* = 0.025), PLR (HR: 165.50, 95% CI: 1.76–15,538.04, *P* = 0.0027) and time in mitotane target (HR: 0.017, 95% CI: 0.001–0.27, *P* = 0.004) maintained a significant association with OS (Table 3). NLR and dNLR were also associated with shorter TTP (HR: 2.58, 95% CI: 1.28–5.20, *P* = 0.008; HR: 2.37, 95% CI: 1.19–4.72, *P* = 0.014, respectively), but only at univariable analysis (Supplementary Table 2).

**Table 3:** MITOTANE COHORT – Univariable and multivariable analysis of clinic-pathological factors predictive of overall survival (OS).

		Univariable analysis			Multivariable analysis		
Variable	Median OS (months)	HR	95% CI	*P*	HR	95% CI	*P*
ENSAT I-II-III	20.00	2.114	0.941–4.752	0.070	3.388	0.041–276.85	0.587
ENSAT IV	7.00						
R-status = 0	20.00	3.446	1.307–9.085	**0.012**	4.890	0.277–86.21	0.278
R-status = X/1/2	10.00						
Ki67< 20	30.00	2.597	0.967–6.975	0.058	6.018	0.184–197.07	0.313
Ki67 ≥ 20	14.00						
Time from diagnosis to start treatment > 6 months	28.00	2.473	1.151–5.313	**0.020**	0.178	0.004–7.371	0.364
Time from diagnosis to start treatment ≤ 6 months	10.00						
ECOG performance status = 0	20.00	2.559	1.159–5.649	**0.020**	1.330	0.028–62.23	0.884
ECOG performance status ≥ 1	9.00						
Cortisol secretion (no)	20.00	2.331	1.028–5.287	**0.043**	0.332	0.034–3.274	0.345
Cortisol secretion (yes)	7.00						
Mitotane in range < 80%	16.00	0.342	0.132–0.885	**0.027**	0.017	0.001–0.271	**0.004**
Mitotane in range ≥ 80%	32.00						
Inflammation-based scores							
NLR < 5	29.00	4.733	2.148–10.428	**< 0.001**	145.834	1.878–11323.83	**0.025**
NLR ≥ 5	7.00						
dNLR < 2.4	32.00	4.683	2.092–10.486	**< 0.001**	0.098	0.006–1.690	0.110
dNLR ≥ 2.4	7.00						
PLR < 190	28.00	3.074	1.404–6.728	**0.005**	165.503	1.763–15538.04	**0.027**
PLR ≥ 190	8.00						
MLR < 0.4	20.00	2.620	1.188–5.777	**0.017**	8.332	0.497–139.61	0.140
MLR ≥ 0.4	10.00						
Albumin > 39 g/L	20.00	1.790	0.819–3.914	0.145	NA	NA	NA
Albumin ≤ 39 g/L	14.00						

Clinical variables with a potential prognostic value at univariate Cox regression (enter level *P* ≤ 0.1) were included in the multivariate Cox model. Significant *P* values are bold. 95% CI, 95% confidence interval; dNLR, derived neutrophil-to-lymphocyte ratio; ECOG, Eastern Cooperative Oncology Group; HR, hazard ratio; MLR, monocyte-to-lymphocyte ratio; NLR, neutrophil-to-lymphocyte ratio; OS, overall survival; PLR, platelet-to-lymphocyte ratio; R-status, resection status.

In the EDP cohort, only NLR (HR: 2.52, 95% CI: 1.31–4.88, *P* = 0.006), dNLR (HR: 1.96, 95% CI: 1.05–3.66, *P* = 0.035), and cortisol hypersecretion (HR: 1.94, 95% CI: 1.02–3.69, *P* = 0.044) predicted OS, although this was not confirmed at multivariable analysis (Table 4). When considering TTP, significant associations were only observed for NLR (HR: 1.95, 95% CI: 1.04–3.66, *P* = 0.037) and dNLR (HR: 2.06, 95% CI: 1.06–4.01, *P* = 0.034), thus multivariable analysis was not performed (Supplementary Table 3).

**Table 4:** EDP COHORT – univariable and multivariable analysis of clinic-pathological factors predictive of overall survival (OS).

		Univariable analysis			Multivariable analysis		
Variable	Median OS (months)	HR	95% CI	*P*	HR	95% CI	*P*
ENSAT I-II-III	10.00	1.286	0.689–2.400	0.430	NA	NA	NA
ENSAT IV	11.00						
R-status = 0	10.00	0.840	0.403–1.753	0.643	NA	NA	NA
R-status = X/1/2	11.00						
Ki67< 20	9.00	0.448	0.165–1.218	0.116	NA	NA	NA
Ki67 ≥ 20	14.00						
Time from diagnosis to start treatment >6 months	10.00	1.446	0.775–2.698	0.247	NA	NA	NA
Time from diagnosis to start treatment ≤6 months	10.00						
ECOG performance status = 0	13.00	1.844	0.903–3.764	0.093	1.880	0.828–4.269	0.131
ECOG performance status ≥ 1	9.00						
Cortisol secretion (no)	13.00	1.940	1.018–3.698	**0.044**	1.400	0.615–3.186	0.423
Cortisol secretion (yes)	10.00						
Mitotane in range< 80%	10.00	1.133	0.519–2.472	0.754	NA	NA	NA
Mitotane in range ≥ 80%	16.00						
Inflammation-based scores							
NLR < 5	14.00	2.524	1.306–4.880	**0.006**	1.995	0.713–5.578	0.188
NLR ≥ 5	9.00						
dNLR < 2.4	14.00	1.960	1.050–3.659	**0.035**	0.867	0.300–2.501	0.791
dNLR ≥ 2.4	9.00						
PLR < 190	17.00	1.768	0.913–3.421	0.091	2.061	0.789–5.388	0.140
PLR ≥ 190	10.00						
MLR < 0.4	13.00	1.334	0.713–2.496	0.368	NA	NA	NA
MLR ≥ 0.4	10.00						
Albumin > 39 g/L	10.00	0.893	0.419–1.905	0.770	NA	NA	NA
Albumin ≤ 39 g/L	9.00						

Clinical variables with a potential prognostic value at univariate Cox regression (enter level *P* ≤ 0.1) were included in the multivariate Cox model; 95% CI, 95% confidence interval; dNLR, derived neutrophil-to-lymphocyte ratio; ECOG, Eastern Cooperative Oncology Group; HR, hazard ratio; MLR, monocyte-to-lymphocyte ratio; NLR, neutrophil-to-lymphocyte ratio; OS, overall survival; PLR, platelet-to-lymphocyte ratio; R-status, resection status.

**Figure 2:**
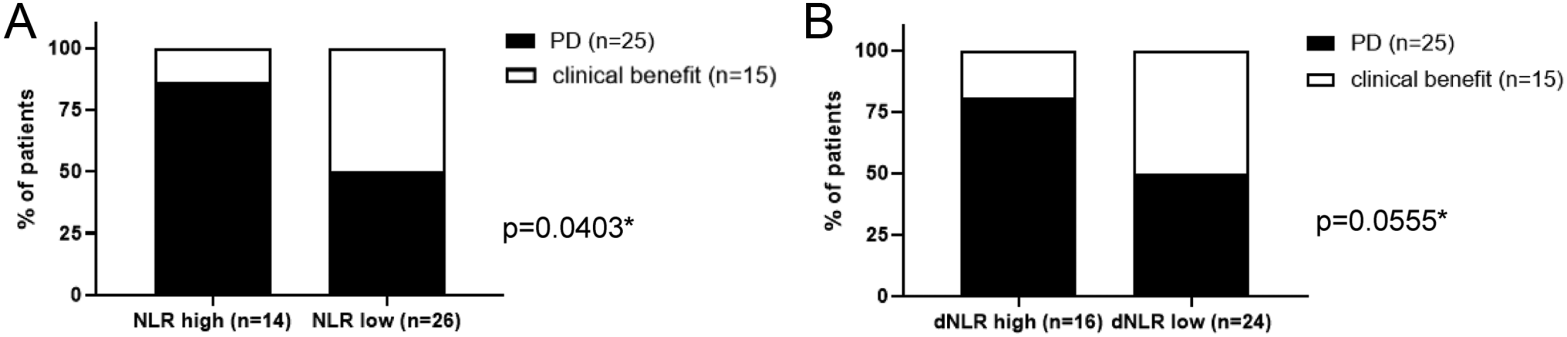
Best objective response in the mitotane cohort (*n* = 40). dNRL, derived neutrophil-to-lymphocyte ratio; NLR, neutrophils-to-lymphocyte ratio; PD, progressive disease. Clinical benefit was defined as stable disease + partial response + complete response + mixed response. Patients were stratified according to pre-treatment NLR (A) and dNLR (B): high (≥5) and low NLR (<5) and high (≥2.5) and low dNLR (<2.5). *P values calculated with absolute values (*P* with % values <0.001) by Chi-square test.

Regarding the objective response to therapy, patients in the mitotane cohort with pre-treatment NLR ≥ 5 showed a worse clinical benefit than those with NLR < 5 (*P* = 0.040) (Fig. 2A). Only two (14.2%) patients with high NLR experienced a clinical benefit from mitotane treatment compared to 50% of those with NLR < 5, including two (7.7%) CRs. Similarly, dNLR ≥ 2.5 tended to predict a worse response to mitotane (*P* = 0.055, Fig. 2B). All other inflammation scores did not reach significance in either of the two cohorts.

## Discussion

In the present study, we investigated for the first time the role of inflammation-based scores in predicting the response to first-line pharmacotherapy in advanced ACC. This is of particular clinical relevance considering that the identification of parameters of treatment response has traditionally failed in ACC due to the rarity of the disease and the low frequency of highly treatment-responsive patients.

Inflammation-based scores reflect cancer-related inflammation, which is proven to affect the tumour microenvironment (de Visser & Coussens 2006, Hanahan & Weinberg 2011) and to be associated with poor prognosis (Stotz *et al.* 2013, Feng *et al.* 2014, Yodying *et al.* 2016). Patients with advanced cancer usually experience a change in peripheral blood cell composition characterized by an expansion of the myeloid components and a reduction of the lymphoid components (Capone *et al.* 2018). Previous studies evaluating immune parameters in adrenal tumours reported significantly higher NLR in ACC than in non-malignant cases (Mochizuki *et al.* 2017, Gaitanidis *et al.* 2019, Sisman *et al.* 2020, Detomas *et al.* 2022). Moreover, studies of preoperative inflammation scores in patients with ACC who underwent resection of the primary tumour demonstrated a prognostic role of both NLR and PLR, although the cut-offs utilized and the results slightly differed between studies (Bagante *et al.* 2015, de Jong *et al.* 2021, Solak *et al.* 2021). Bagante and colleagues also underlined how NLR and PLR were associated with larger tumours, suggesting a more aggressive behaviour (Bagante *et al.* 2015). Furthermore, Grisanti *et al.* demonstrated NLR ≥ 5 to be a strong independent indicator of progression-free survival in patients with metastatic ACC treated with gemcitabine plus capecitabine (Grisanti *et al.* 2021).

In the present study, we demonstrated the prognostic role of NLR and PLR in patients receiving mitotane as palliative therapy. Both NLR and PLR were good predictors of longer OS and TTP – the last being significant only at univariable analysis – when pretreatment values were lower than the used cut-offs. On the contrary, traditional clinical prognostic values failed to maintain significance at multivariable analysis, except for plasma mitotane levels within the target range. Additionally, low NLR predicted a better objective response to mitotane.

Pretreatment serum albumin, which has been proposed as a prognostic factor for patients with ACC after primary resection (Zhang *et al.* 2021), did not show a predictive role in our study. With respect to dNLR, although it showed an association with outcomes at univariable analysis, it proved to be less robust than NLR in predicting both OS and response to therapy. Because the two scores are similarly derived from WBCD, we may conclude it to be a redundant parameter if NLR is already calculated.

In the EDP cohort, the predictive role of inflammation-based scores was less evident. This could be due to the difference in patient selection as mitotane monotherapy is usually proposed for patients with lower tumour burden or slower growth, whilst EDP is preferred in more advanced and disseminated disease. In fact, our EDP cohort had adverse clinical characteristics at the time of tumour diagnosis, with half of the patients presenting with metastatic disease, a higher median Ki67, and a worse ECOG performance status. Moreover, some of our patients started EDP after showing tumour progression under mitotane. Therefore, it is not surprising that both OS and TTP proved to be worse in our EDP cohort, hence making it difficult to detect therapy’s effects in a shorter follow-up time. Moreover, the EDP ± mitotane regimen is burdened with considerable toxicity and a significant percentage of our patients were treated with decreased dosages of etoposide and/or doxorubicin, or discontinued treatment because of adverse events (Turla *et al.* 2022). Therefore, in the setting of advanced cancer with such limited treatment options, a more personalized approach geared to preserving the quality of life of patients is of the uttermost importance (Steenaard *et al.* 2019).

Our findings are only partially in agreement with the ones showed by Grisanti and colleagues on the role of NLR in predicting response to second-line treatment with gemcitabine plus capecitabine (Grisanti *et al.* 2021), as NLR was useful in predicting progression and not survival in their study. However, our results are not fully comparable because of the different chemotherapy regimens and outcome measures.

Further interest in inflammatory parameters in ACC derives from the impact of endogenous hormonal secretion and/or exogenous steroid replacement therapy. Cortisol excess can affect white blood cell count and especially NLR and dNLR, due to its effect in increasing the neutrophils (Detomas *et al.* 2022). We assessed the presence of cortisol hypersecretion at treatment initiation, which was associated with worse survival, yet this was not confirmed at multivariable regression. Previous studies have pointed out how cortisol-secreting ACCs are associated with worse outcomes; however, it is not clear whether this is due to the known negative effects of cortisol action or indicates a more aggressive ACC subtype (Vanbrabant *et al.* 2018). In our cohort of patients with advanced ACC, cortisol secretion was particularly difficult to assess due to the frequent ongoing chronic treatment with mitotane, which inhibits steroidogenesis. Moreover, ACC is known to be able to produce precursors and metabolites as well as end-products of steroidogenesis, thus even ACC considered as non-functioning could potentially have a subclinical production thus impacting blood counts (Arlt *et al.* 2011, Bancos *et al.* 2020).

The present study harbours some limitations due to its retrospective nature and the relatively small number of patients included, because of the rarity of ACC. However, our data on the significant and independent role of NLR in predicting the response to treatment in patients with advanced ACC represent a promising starting point for subsequent multicentre studies. The future validation of these biomarkers in larger cohorts would offer significant clinical benefits, as inflammation-based scores could represent a non-invasive, inexpensive, and easy-to-use tool to predict outcomes in patients with advanced ACC. As NLR and PLR could be easily translated to clinical practice, these biomarkers might be taken into consideration during therapeutic decision-making in advanced ACC, to try to avoid toxicity in patients who are not likely to benefit from chemotherapy.

## Supplementary Material

Supplementary Table 1. Demographic and clinical data of 90 patients with advanced adrenocortical carcinoma (ACC) at initial diagnosis

Supplementary Table 2 - MITOTANE COHORT – Univariable and multivariable analysis of clinic-pathological factors predictive of time-to progression (TTP).

Supplementary Table 3 - EDP COHORT - Univariable analysis of clinic-pathological factors predictive of time-to progression (TTP).

## Declaration of interest

The authors declare that there is no conflict of interest that could be perceived as prejudicing the impartiality of the research reported.

## Funding

This work was partially supported by the Deutsche Krebshilfe Stifftung (project number 70112969 to CLR), the Association for Multiple Endocrine Neoplasia Disorders (AMEND) (ACC Research Award 2021 to CLR), the European Reference networks Endo-ERN and EuRanCan, and Ricerca Corrente Funds from the Italian Ministry of Health to Fondazione IRCCS Ca’ Granda Ospedale Maggiore Policlinico (to GM).
