# Supplementary material for: Inflammation-based scores as predictors of treatment response in advanced adrenocortical carcinoma
Source: Endocr Relat Cancer. 2023 Mar 15;30(4):e220372. doi: 10.1530/ERC-22-0372 (PMC10083578; doi:10.1530/ERC-22-0372)
Supplement: Supplementary Table 1. Demographic and clinical data of 90 patients with advanced adrenocortical carcinoma (ACC) at initial diagnosis [file supplementary_table_1.pdf]

Supplementary Table 1. Demographic and clinical data of 90 patients with advanced adrenocortical carcinoma (ACC) at initial diagnosis

At initial diagnosis	Mitotane cohort	EDP cohort	p value
Cases, n	40	50*	
Sex, F/M (% F)	24/16 (60)	29/21 (58)	1.000
Median age (range), years	52.5 (23-83)	49.5 (20-77)	0.114
Symptoms related to ACC (%)	34/40 (85)	44/50 (88)	0.760
ENSAT tumor stage:			
II (%)	14/40 (35)	12/50 (24)	0.214
III (%)	14/40 (35)	14/50 (28)	
IV (%)	12/40 (30)	24/50 (48)	
Median Ki67 (range), %	20 (1-50)	27 (2-80)	**0.015**
<20 (%)	13/29 (45)	5/35 (14)	**0.011**
≥20 (%)	16/29 (55)	30/35 (86)	
Unknown	11/40	15/50	
Primary surgery not performed (%)	4/40 (10)	14/50 (28)	**0.038**
Resection status:			
R0 (%)	19/36 (53)	18/36 (50)	0.182
RX (%)	1/36 (3)	3/36 (8)	
R1 (%)	9/36 (25)	4/36 (11)	
R2 (%)	4/36 (11)	9/36 (25)	
Unknown	3/36 (8)	2/36 (6)	

Abbreviations: EDP, etoposide, doxorubicin and cisplatin; N, number; F, female; M, male; ACC, adrenocortical carcinoma; R, resection status.
