# Supplementary material for: Inflammation-based scores as predictors of treatment response in advanced adrenocortical carcinoma
Source: Endocr Relat Cancer. 2023 Mar 15;30(4):e220372. doi: 10.1530/ERC-22-0372 (PMC10083578; doi:10.1530/ERC-22-0372)
Supplement: Supplementary Table 2 - MITOTANE COHORT – Univariable and multivariable analysis of clinic-pathological factors predictive of time-to progression (TTP). [file supplementary_table_2.pdf]

Supplementary Table 2 - MITOTANE COHORT – Univariable and multivariable analysis of clinic-pathological factors predictive of time-to progression (TTP).

		Univariable analysis			Multivariable analysis		
**Variable**	**Median TTP (mo)**	**HR**	**95% CI**	**p**	**HR**	**95% CI**	**p**
ENSAT at diagnosis I-II-III	5.00	1.863	0.878-3.957	0.105	NA	NA	NA
ENSAT at diagnosis IV	2.00						
R-status =0	5.00	2.245	0.968-5.210	0.060	2.355	0.602-9.218	0.129
R-status =X/1/2	3.00						
Ki67<20	14.00	4.862	1.759-13.44	**0.002**	5.758	1.314-25.236	**0.020**
Ki67 ≥20	3.00						
Time from diagnosis to start treatment >6 months	5.00	2.000	0.999-4.001	**0.050**	0.883	0.109-7.127	0.907
Time from diagnosis to start treatment ≤6 months	3.00						
ECOG Performance Status =0	6.00	2.299	1.119-4.726	**0.024**	1.872	0.508-6.899	0.346
ECOG Performance Status ≥1	3.00						
Cortisol secretion (no)	5.00	1.278	0.590-2.767	0.534	NA	NA	NA
Cortisol secretion (yes)	4.00						
Mitotane in range<80%	4.00	0.449	0.201-1.006	0.052	0.162	0.046-0.568	**0.004**
Mitotane in range≥80%	9.00						
**Inflammation-based scores**							
NLR <5	5.00	2.583	1.283-5.202	**0.008**	5.111	0.632-41.331	0.126
NLR ≥5	2.00						
dNLR <2.4	5.00	2.372	1.192-4.723	**0.014**	0.309	0.051-1.857	0.199
dNLR ≥2.4	3.00						
PLR <190	5.00	1.886	0.954-3.730	0.068	2.967	0.321-27.463	0.338
PLR ≥190	3.00						
MLR <0.4	4.00	1.181	0.601-2.320	0.630	NA	NA	NA

MLR ≥0.4	4.00						
Albumin >39 g/L	5.00	1.739	0.851-3.551	0.129	NA	NA	NA
Albumin ≤39 g/L	4.00						

Abbreviations: TTP, time-to-progression; HR, hazard ratio; 95% CI, 95% confidence interval; R-status, resection status; ECOG, Eastern Cooperative Oncology Group; NLR, neutrophil-to-lymphocyte-ratio; dNLR, derived neutrophil-to-lymphocyte ratio; PLR, platelet-to-lymphocyte-ratio; MLR, monocyte-to-lymphocyte-ratio.

Clinical variables with a potential prognostic value at univariate Cox regression (enter level p ≤0.1) were included in the multivariate Cox model.
