# Supplementary material for: Inflammation-based scores as predictors of treatment response in advanced adrenocortical carcinoma
Source: Endocr Relat Cancer. 2023 Mar 15;30(4):e220372. doi: 10.1530/ERC-22-0372 (PMC10083578; doi:10.1530/ERC-22-0372)
Supplement: Supplementary Table 3 - EDP COHORT - Univariable analysis of clinic-pathological factors predictive of time-to progression (TTP). [file supplementary_table_3.pdf]

Supplementary Table 3 - EDP COHORT - Univariable analysis of clinic-pathological factors predictive of time-to progression (TTP).

		Univariable analysis		
**Variable**	**Median TTP (mo)**	**HR**	**95% CI**	**p**
ENSAT at diagnosis I-II-III	3.00	0.933	0.504-1.729	0.826
ENSAT at diagnosis IV	4.00			
R-status =0	3.00	0.754	0.337-1.684	0.491
R-status =X/1/2	4.00			
Ki67<20	4.00	2.268	0.529-9.715	0.270
Ki67 ≥20	3.00			
Time from diagnosis to start treatment >6 months	3.00	1.125	0.579-2.185	0.728
Time from diagnosis to start treatment ≤6 months	4.00			
ECOG Performance Status =0	3.00	1.110	0.556-2.217	0.767
ECOG Performance Status ≥1	4.00			
Cortisol secretion (no)	3.00	1.201	0.646-2.233	0.562
Cortisol secretion (yes)	4.00			
Mitotane in range<80%	3.00	0.973	0.433-2.203	0.954
Mitotane in range≥80%	4.00			
**Inflammation-based scores**				
NLR <5	6.00	1.955	1.043-3.663	**0.037**
NLR ≥5	3.00			
dNLR <2.4	6.00	2.060	1.057-4.012	**0.034**
dNLR ≥2.4	3.00			
PLR <190	6.00	1.915	0.953-3846	0.068
PLR ≥190	3.00			

MLR <0.4	6.00	1.644	0.835-3.238	0.150
MLR ≥0.4	3.00			
Albumin >39 g/L	3.00	1.233	0.520-2.921	0.635
Albumin ≤39 g/L	3.00			

Abbreviations: TTP, time-to-progression; HR, hazard ratio; 95% CI, 95% confidence interval; R-status, resection status; ECOG, Eastern Cooperative Oncology Group; NLR, neutrophil-to-lymphocyte-ratio; dNLR, derived neutrophil-to-lymphocyte ratio; PLR, platelet-to-lymphocyte-ratio; MLR, monocyte-to-lymphocyte-ratio.
